# Effect of shock wave power spectrum on the inner ear pathophysiology in blast-induced hearing loss

**DOI:** 10.1038/s41598-021-94080-0

**Published:** 2021-07-19

**Authors:** Eiko Kimura, Kunio Mizutari, Takaomi Kurioka, Satoko Kawauchi, Yasushi Satoh, Shunichi Sato, Akihiro Shiotani

**Affiliations:** 1 Department of Otolaryngology-Head and Neck Surgery, National Defense Medical College, 3-2 Namiki, Tokorozawa, Saitama, 359-8513 Japan; 2 Division of Bioinformation and Therapeutic Systems, National Defense Medical College Research Institute, Saitama, 359-8513 Japan; 3 Department of Biochemistry, National Defense Medical College, Saitama, 359-8513 Japan

**Keywords:** Neuroscience, Medical research

## Abstract

Blast exposure can induce various types of hearing impairment, including permanent hearing loss, tinnitus, and hyperacusis. Herein, we conducted a detailed investigation of the cochlear pathophysiology in blast-induced hearing loss in mice using two blasts with different characteristics: a low-frequency dominant blast generated by a shock tube and a high-frequency dominant shock wave generated by laser irradiation (laser-induced shock wave). The pattern of sensorineural hearing loss (SNHL) was low-frequency- and high-frequency-dominant in response to the low- and high-frequency blasts, respectively. Pathological examination revealed that cochlear synaptopathy was the most frequent cochlear pathology after blast exposure, which involved synapse loss in the inner hair cells without hair cell loss, depending on the power spectrum of the blast. This pathological change completely reflected the physiological analysis of wave I amplitude using auditory brainstem responses. Stereociliary bundle disruption in the outer hair cells was also dependent on the blast’s power spectrum. Therefore, we demonstrated that the dominant frequency of the blast power spectrum was the principal factor determining the region of cochlear damage. We believe that the presenting models would be valuable both in blast research and the investigation of various types of hearing loss whose pathogenesis involves cochlear synaptopathy.

## Introduction

The necessity for research on blast-induced hearing impairment has escalated, as the ear is the most vulnerable organ in the event of blast exposure^1,2^, thus becoming an increasingly common casualty in military and civilian situations^3,4^. Blast exposure causes various types of pathologies in the external, middle, and inner ear^5,6^. The most common feature of blast exposure is tympanic membrane perforation (TMP)^7,8^. Temporary and permanent hearing loss, tinnitus, and hyperacusis are also among the common signs of blast exposure^9,10^. Although permanent sensorineural hearing loss (SNHL) is currently untreatable, middle ear pathologies, including TMP, can be treated even though they require surgery. Moreover, permanent SNHL caused by blast exposure is often associated with a decline in the quality of life^11,12^. Therefore, blast-induced SNHL animal models have been established by various studies to investigate the detailed mechanisms of this pathology^13–17^. Conventionally, blast injury models generated using a blast tube, which consists of an air-compressed tube structure, have been used to study traumatic brain injury (TBI)^14^. However, since the blast intensity required to induce a permanent threshold shift (PTS) is relatively high, adjusting the intensity to induce a PTS without fatal TBI using the conventional chamber is a difficult task. Moreover, the blast intensity required to generate a PTS is usually strong enough to induce conductive hearing loss, such as TMP, which makes it impossible to accurately evaluate the inner ear functions using auditory brainstem responses (ABRs) or distortion-product otoacoustic emissions (DPOAEs). Multiple exposures to low-intensity blasts were also used in an attempt to generate pure SNHL without TMP^15–17^. However, a single exposure would be ideal to investigate the relationship between the property of the shock wave and the degree of inner ear damage.

A mouse model is useful for detailed pathological analyses because of its small size, cost-effectiveness, and availability of transgenic animals. Hence, we developed two different types of blast-induced SNHL mouse models. The first was created using a conventional shock tube, which consists of the driver (high-pressure) and driven sections separated by a diaphragm^18^. This model was originally used to create blast-induced mild traumatic injury (bmTBI) using single shock-wave irradiation; therefore, the intensity of the irradiated shock wave is relatively mild. We induced mild SNHL without TMP in our preliminary experiment. The other model of unilateral hearing loss and tinnitus was generated via irradiation with a laser-induced shock wave (LISW)^19^, which is our unique blast injury model of pure unilateral hearing loss and tinnitus following unilateral cochlear damage^20^. Furthermore, this model was reported to have good reproducibility for generating mild SNHL without TMP^19^. The advantage of these two models for hearing-related research is that they do not cause damage to the middle ear (including TMP), making it possible to investigate pure SNHL.

Using these two blast-induced SNHL mouse models, this study aimed to analyze the relationship between the property of the blast shock wave and the pathophysiological effect on the inner ear after blast exposure. Originally, we utilized the LISW model as blast exposure with ear protection, which is a common situation for soldiers on the battlefield, as LISW propagates into the inner ear via bone conduction, a condition similar to blast exposure with ear protection. The shock tube approach models a mix of air- and bone-conduction exposure, similar to human blast exposures without hearing protection. The intensities of shock wave were adjusted to induce almost the same severity of damage as that at 16 kHz, a low-hearing-threshold frequency in mice^21,22^, to facilitate the comparison of the effect of the two different blasts on the inner ear. We investigated the relationship between the site of inner ear dysfunction physiologically and anatomically and the peak pressure of the exposed shock wave.

## Results

### Two different shock wave generators created shock waves with distinct properties

**Figure 1:**
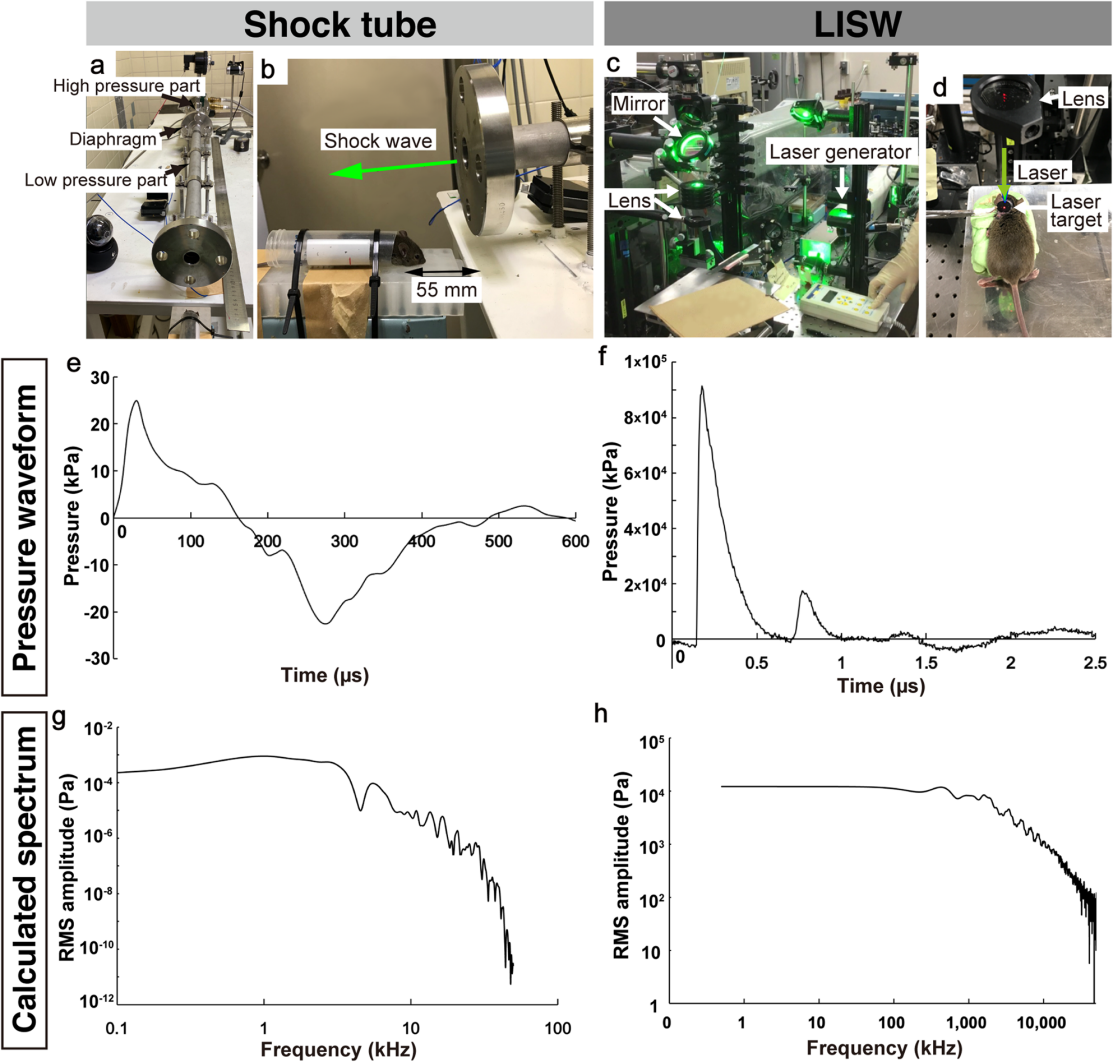
Appearance of the shock-wave generators and characteristics of the shock wave used in the experiments. (**a**, **b**) Experimental setup of the shock tube. The stainless steel tubing (inner diameter: 25 mm, outer diameter: 34 mm) was divided by a polyester diaphragm into high- and low-pressure parts (length of low-pressure part: 800 mm, high-pressure part: 400 mm). The mouse was placed 55 mm diagonally beneath the edge of the tube (**c**, **d**). Experimental setup for the LISW. Shock waves were generated by irradiating a laser target with a Q-switched Nd:YAG laser. Plasma formation occurred at the interface of the laser target, and its expansion was accompanied by the generation and LISW, which was irradiated onto the inner ear through the skin (**e**, **f**). Typical temporal pressure waveforms of the shock wave generated by the shock tube as measured at the tragus (**e**), and LISW (**f**). Typical negative pressure followed by positive peak pressure was clearly reproduced by the shock tube (**e**). LISW can generate intense positive peak pressure with a considerably shorter duration compared to the shock tube (**f**). (**g**, **h**) Pressure frequency spectra were obtained from the pressure waveforms of the shock waves spanning the range of peak pressures using fast Fourier transform. While the respective forms of each spectrum were similar, the frequency range of the shock wave generated by the shock tube (x-axis: frequency) (**g**) was substantially narrower and lower than that in the LISW (**h**). LISW, laser-induced shock wave. RMS, root mean squared.

We used two different types of blast-induced hearing loss mouse models in the present study. The waveform of the shock wave generated by the shock-tube system used in this study showed a steep rise in the positive peak with 25.0 kPa (Fig. 1e). The duration of the positive pressure part of the shock wave was 162 µs. The impulse of this shock wave was 1.14 Pa.s. On the other hand, the waveform of the LISW used in this study showed a steep rise with a positive peak pressure of 91.3 MPa, which was a thousand times higher than that obtained by the shock tube (Fig. 1f). The duration of the positive pressure was 1.1 µs, which was considerably shorter than that obtained by the shock tube. The impulse of this shock wave was 14.6 Pa.s.

Moreover, we calculated the frequency spectra from the observed pressure waveforms using fast Fourier transform. The frequency of the median root mean squared (RMS) amplitude of the shock tube-induced shock wave was 976.5 Hz, whereas the frequency of the median RMS amplitude of the LISW was 448 kHz (500 times higher than that of the shock tube induced shock wave). Moreover, the LISW consisted of a substantially higher range of frequencies compared to the shock tube induced shock wave.

### The degree of hearing impairment after blast exposure was determined by the frequency spectrum of the exposed shock wave

We observed the tympanic membrane in all cases immediately after blast exposure before conducting the hearing assessment. We did not observe TMP in the mice after blast exposure in any group (Supplemental Fig. 1). Furthermore, mechanical damage was not observed in the cochleae, including round window rupture, stapes dislocation, intracochlear hemorrhage, and basilar or Reissner membrane rupture, under light microscopy using frozen cross-sections after LISW exposure (data not shown). Therefore, the hearing impairment observed in all groups in this study was diagnosed as SNHL.

**Figure 2:**
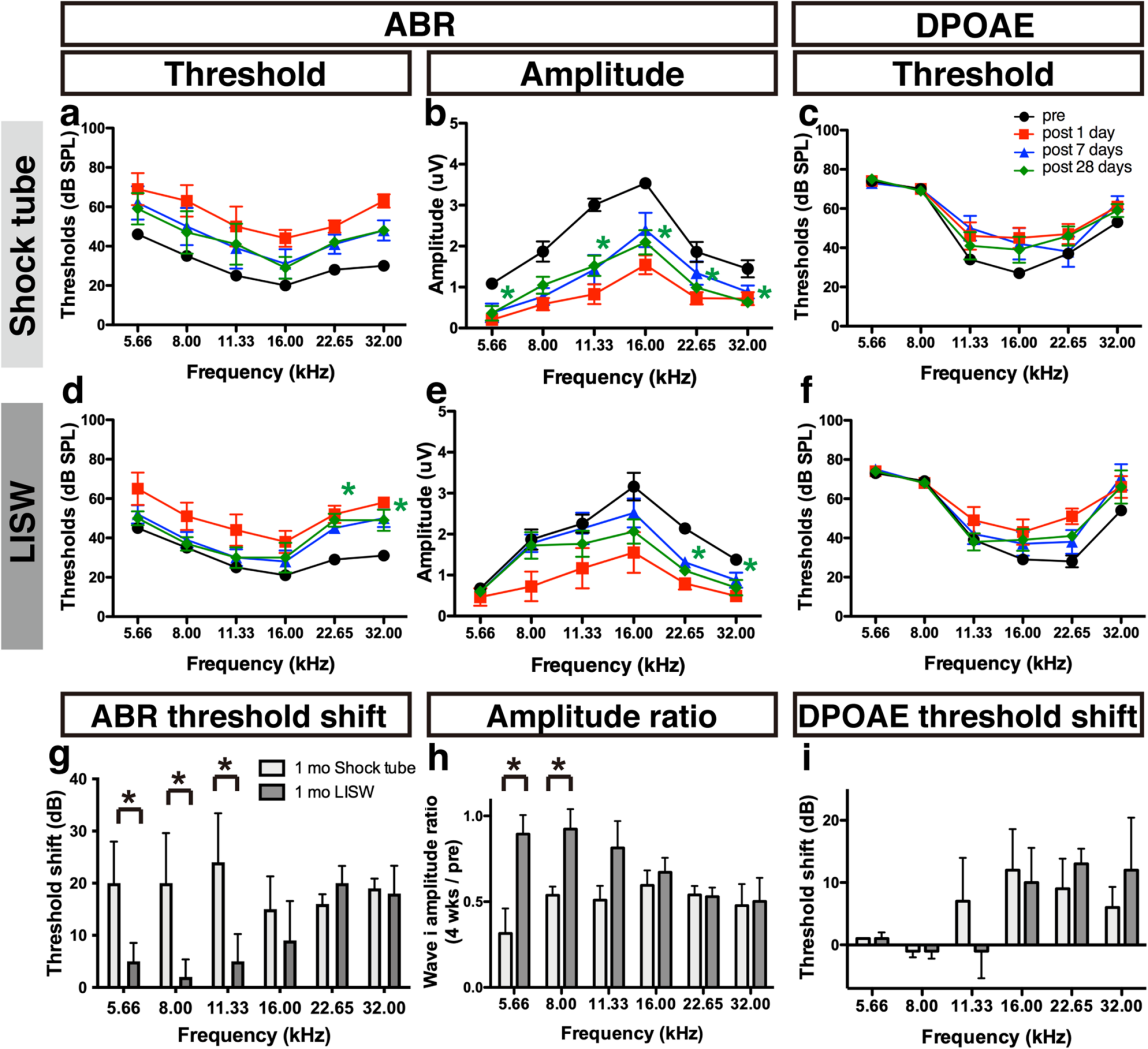
Measurement of hearing function using ABR and DPOAE before and after shock-wave exposure. (**a**, **d**) Significant ABR threshold shifts were observed one day after shock-wave exposure (filled red squares) for up to 28 days after exposure (filled green diamonds) at higher frequencies in the LISW group (**d**) compared to the pre-exposure thresholds (filled circles). Although the thresholds 28 days after exposure at all frequencies were larger than the pre-exposure thresholds, no significant elevation of the ABR threshold was observed in the shock-tube group (**a**). (**b**, **e**) The ABR wave I amplitude was decreased at all frequencies in the shock-tube group, (**b**) which was also seen in the LISW group, at the same frequencies at which the threshold shift was observed (**e**). (**c**, **f**) A temporal decrease of DPOAE level was observed at 1 day after exposure both in shock tube and LISW group. Although the DPOAE levels 28 days after exposure at higher frequencies were larger compared to the pre-exposure levels, no significant elevation of the DPOAE level was observed at 28 days after both in shock tube and LISW group (**g**) The ABR threshold shifts (the threshold at 28 days after exposure minus that at pre-exposure) were significantly higher in the shock-tube group at the lower frequencies (5.55–11.33 kHz). (**h**) The ratio of the wave I amplitude (28 days after exposure to pre-exposure) also decreased in both the shock-tube and LISW groups at all frequencies. Significant differences were observed at the lower frequencies (5.66 and 8.00 kHz). (**i**) The DPOAE threshold shift between the shock tube and LISW group 28 days after exposure did not differ significantly. The asterisks indicate significant differences (*p* < 0.05) compared to the pre-exposure values (2-way ANOVA, followed by Bonferroni correction for multiple comparisons). The error bars indicate the SEs of the means (n = 5 in each group). ABR, auditory brainstem response; DPOAE, distortion product otoacoustic emission; LISW, laser-induced shock wave; SE, standard error.

First, we conducted several complementary tests to assess inner ear function using the ABR threshold, ABR wave I amplitude, and DPOAE measurements. The ABR is a sound-evoked potential in the ascending auditory pathways, and the first ABR wave (wave I) represents the summed activity of the cochlear nerve^23^. The DPOAE can directly measure the function of the outer hair cell (OHC), which serves as a biological motor for amplifying the motion of the sensory epithelium^24^. The shock-tube group exhibited a remarkable elevation in the ABR threshold one day after the shock wave exposure at all tested frequencies, but the ABR threshold was not significantly elevated 28 days after exposure compared to that at pre-exposure, although the thresholds 28 days after exposure at all frequencies were larger than the pre-exposure thresholds (Fig. 2a). The amplitude of the ABR wave I decreased one day after shock-wave exposure at all tested frequencies. Unlike the ABR threshold, the decrease in ABR wave I amplitude was sustained for up to 28 days after blast exposure at almost all frequencies (2-way ANOVA, F_4, 120_ = 29.23, *p* < 0.001) (Fig. 2b). The trend in the changes in the DPOAE threshold before and after blast exposure closely resembled that in the ABR threshold (Fig. 2c).

A remarkable elevation in the ABR threshold was also observed one day after shock-wave exposure at all frequencies in the LISW group. Moreover, the ABR thresholds 28 days after exposure were significantly elevated at 22.65 and 32.00 kHz compared to those at pre-exposure (2-way ANOVA, F_4, 120_ = 14.86, *p* < 0.001; Bonferroni multiple comparison significance at 22.65 kHz [*p* = 0.025], at 32.00 kHz [*p* = 0.040], respectively) (Fig. 2a). The ABR wave I amplitude decreased one day after the shock wave exposure at all frequencies. Interestingly, the decrease in ABR wave I amplitude was sustained only at higher frequencies: Significant differences were observed in the ABR threshold up to 28 days after blast exposure compared to those at pre-exposure (2-way ANOVA, F_4, 120_ = 13.11, *p* < 0.001; Bonferroni multiple comparison significance at 22.65 kHz [*p* = 0.026], at 32.00 kHz [*p* = 0.031], respectively) (Fig. 2e). The trend in the DPOAE threshold changes in the LISW group closely resembled that in the shock-tube group (Fig. 2f).

The two models were compared directly by calculating the “threshold shift” i.e., the ABR/DPOAE threshold obtained 28 days after exposure was subtracted from that obtained before exposure, and the “amplitude ratio,” which was calculated by dividing the ABR wave I amplitude value obtained 28 days after exposure from that obtained before exposure. The comparison of “threshold shift” in the ABR proved that the shift caused by the shock tube was dominant at lower frequencies; in contrast, the shift caused by LISW was dominant at higher frequencies (Fig. 2g). The trend in the ratio of the ABR wave I amplitude was similar to that in the ABR threshold shift (Fig. 2h). The trend of damage dominance in the DPOAE threshold shift was ambiguous, as a significant increase in the shift in the shock-tube model was not observed at any frequency (2-tailed unpaired Student’s *t-*test) (Fig. 2i).

### The blast reduced the number of synapses in the inner hair cells (IHCs) but did not induce hair cell loss

**Figure 3:**
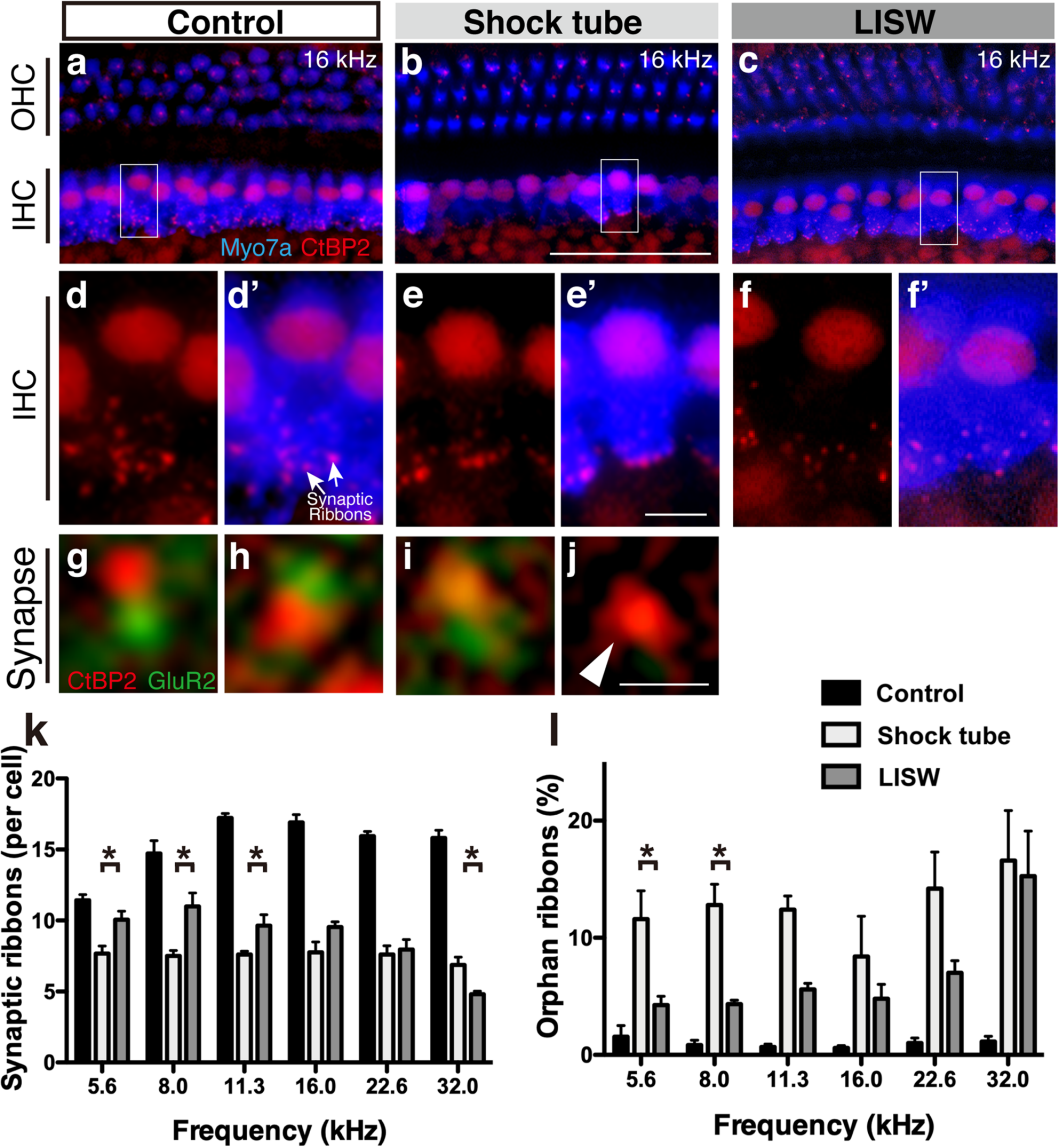
Changes in the organ of Corti after shock-wave exposure. (**a**–**f**) Confocal image of immunohistochemical analysis of the organs of Corti (**a**–**c**, blue, anti-myosin 7a; red, CtBP2), enlarged image of the inner hair cells seen in the white square in **a**–**c** (**d**–**f**, blue, anti-myosin 7a; red, anti-CtBP2; anti-CtBP2 also stains the IHC nuclei) at 16 kHz. (**g**–**j**) A typical example of synaptic complexes from the non-exposed (**g**, **h**) and shockwave-exposed (**i**, **j**) z-stacks, red, anti-CtBP2; green: anti-GluA2. Orphan ribbons, lacking apposed glutamate receptor patches, were observed in the blast-exposed ears (**j**). The white arrowhead indicates orphan ribbons (**k**). The quantification of the synaptic ribbons from a single IHC, observed 28 days after exposure and in the controls. The number of synaptic ribbons decreased in both the shock-tube and LISW groups compared to the control group at all tested frequencies. The number of synaptic ribbons was significantly fewer in the shock-tube group at the lower frequencies (5.6 and 8.0 kHz) and fewer in the LISW group at a higher frequency (32.0 kHz): these trends were similar to the ABR threshold shift shown in Fig. 2g. (**l**) The histograms compare the % of orphan ribbons per single IHC at the frequencies at which the ABR and DPOAE were measured, as compiled for all control and exposed (shock tube and LISW) cases. All data were gathered 28 days post-blast exposure. The asterisks indicate significant differences (*p* < 0.05) (2-way ANOVA, followed by Bonferroni correction for multiple comparisons). The error bars indicate the SEs of the means (n = 5 in each group). The scale bar represents 5 μm. The scale bar in panel b, which represents 50 µm, is applicable for panels **a**–**c**. The scale bar in panel **e**’, which is 5 µm, is applicable from panels **d**–**f**, and **d**’–**f**’. The scale bar in panel **j**, which represents 0.5 µm, applies from panels **g**–**j**. ABR, auditory brainstem response; DPOAE, distortion product otoacoustic emission; IHC, inner hair cell; LISW, laser-induced shock wave; OHC, outer hair cell.

We subsequently conducted surface preparation analysis of the organ of Corti 28 days after blast exposure to assess the cause of SNHL after blast exposure. The number of OHCs (anti-myosin 7a: blue, Fig. 3a–i) in the blast-exposed ear did not change at any frequency region in any group (Supplemental Fig. 2b), although the ABR threshold was elevated at higher frequencies in the LISW groups (Fig. 2d). The number of IHCs did not change in any group (Supplemental Fig. 2a).

Subsequently, we focused on the synapses between the IHCs and cochlear nerve fibers. Synaptic ribbons are a characteristic structure of hair cell afferent synapses that are involved in vesicle delivery to the active zone^25^. The number of synaptic ribbons (anti-CtBP2: red, Fig. 3a–f) in the shock tube and LISW groups decreased significantly compared to the control group at all frequencies tested (2-way ANOVA, F_2, 72_ = 315.9, *p* < 0.001) (Fig. 3k). The number of synaptic ribbons was significantly lower in the shock-tube group at the lower frequencies (Bonferroni multiple comparison significance at 5.6 kHz [*p* = 0.014], at 8.0 kHz [*p* < 0.001], and at 11.3 kHz [*p* = 0.046]), and fewer in the LISW group at a higher frequency (Bonferroni multiple comparison significance at 32.0 kHz [*p* = 0.042]): these trends were similar to those of the ABR wave I amplitude. We also counted the number of orphan ribbons, i.e., synapse structures lacking apposed glutamate receptor patches (green: anti-GluA2), in the blast-exposed ears (Fig. 3g–j). Similar trends were observed in the number of synaptic ribbons and orphan synapses: The number of orphan synapses was significantly higher in the shock-tube group at the lower frequencies (2-way ANOVA, F_2, 56_ = 35.04, *p* < 0.001; Bonferroni multiple comparison significance at 5.6 kHz [*p* = 0.048], at 8.0 kHz [*p* < 0.035]).

We further analyzed the SGCs 28 days after blast exposure using frozen cross-sections. The number of SGCs in the blast-exposed ear did not change in any cochlear region in any group (Supplemental Fig. 3d).

### The degree of stereociliary bundle disruption in the OHCs was commensurate with the power spectrum of the exposed shock wave

**Figure 4:**
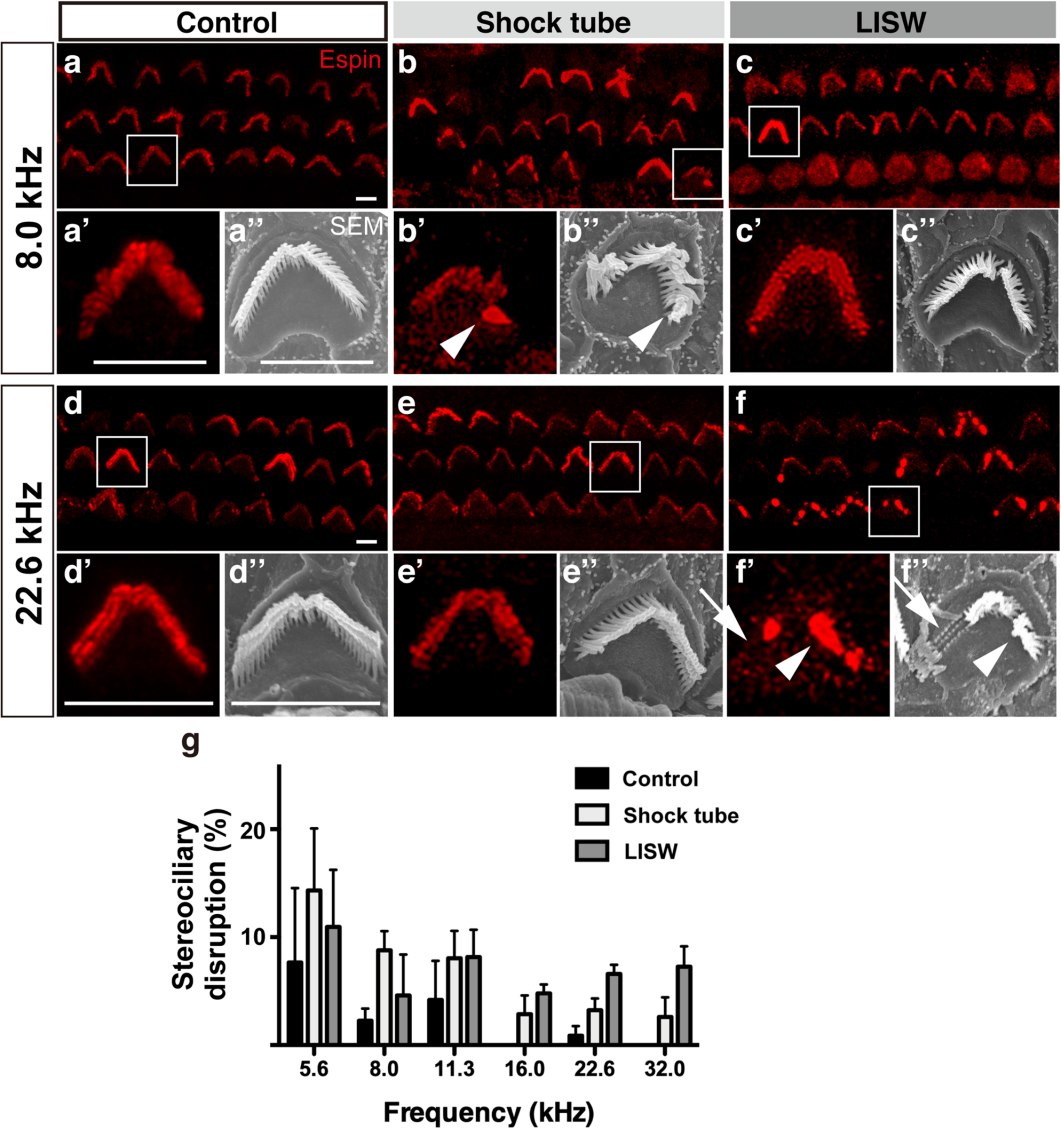
Surface structures in the OHCs. (**a**–**f**) Confocal image of immunohistochemical analysis for three rows of OHC stereocilia (red, anti-espin) at 8.0 kHz (**a**–**c**), and 22.6 kHz. (**d**–**f**) Organ of Corti area 28 days after blast exposure. (**a**’–**f**’) High-power views of stereocilia from a single OHC is shown in the white square (**a**–**f**). (**a**’’–**f**’’) High-power views of stereocilia from a single OHC observed under SEM. The white arrowheads indicate aggregation of stereocilia (**b**’, **b**’’, **f**’, and **f**’’), and the white arrows indicate the partially missing stereocilia. (**g**) Stereociliary disruption ratio in the OHCs 28 days after exposure (n = 5 in each group). The amount of stereociliary disruption was higher in the shock-tube group at the lower frequencies and higher in the LISW group at the higher frequencies (22.6 and 32.00 kHz). The scale bar represents 5 μm. The error bars indicate the SEs of the means (n = 5 in each group). LISW: laser-induced shock wave; OHC: outer hair cell; SE: standard error; SEM: scanning electron microscopy.

We observed that the ABR threshold was elevated up to 28 days after the above-mentioned blast exposure. However, the ABR threshold shift cannot simply be explained by disordered synapses between the IHCs and SGCs or the histological reduction in the number of SGCs if the numbers of IHCs or OHCs are preserved^26^. In a previous study, we found that stereociliary bundle disruption in the OHCs was the primary cause of the ABR threshold shift after LISW exposure in rats^19^. Therefore, we finally conducted a detailed observation and quantification of the surface structure of the OHCs to determine the etiology of ABR threshold elevation. The normal stereocilia in the OHC are “V” shaped and contain three bundle rows (Fig. 4a’, a”, d’, d”). However, deformed stereocilia, which were bent, tangled (Fig. 4b’, b”, white arrowhead), or lacking a part of the bundle (Fig. 4f’, 4f.” white arrow), were observed after blast exposure. Quantitative analysis of stereociliary bundle disruption of the OHCs revealed that the disruption ratio tended to be higher in the LISW group in the higher-frequency area; however, no statistically significant difference was observed among the groups (2-way ANOVA, F_2, 56_ = 3.981, *p* = 0.025; no significant differences in Bonferroni multiple comparison, Fig. 4g). After all, the pattern of distribution of the stereociliary bundle disruption ratio was similar to the trend in the power spectrum of the exposed shock wave shown in Fig. 1. Moreover, the pattern of distribution of the stereociliary bundle disruption ratio closely resembled the trend in the ABR threshold shift (Fig. 2g).

## Discussion

### Power spectrum of the shock wave directly reflects the pattern of inner ear damage after blast exposure

Our analysis of the longitudinal pattern of cochlear damage after blast exposure clearly showed that the degree of the inner ear dysfunction measured using the ratio of ABR changes was directly associated with the frequency distribution of the power spectrum of the exposed shockwave, irrespective of the shock wave generator. The primary site of cochlear damage is determined by the frequency spectrum of the exposed noise in noise-induced hearing loss^27^. The damage was mainly observed in the basal cochlear region on exposure to high-frequency dominant noise, which receives sound of higher frequency, whereas the whole cochlear area was uniformly damaged on exposure to low-frequency noise^28^. A highly similar pattern of cochlear damage was observed in our blast-exposed mice physiologically and histologically. The pattern of degeneration of the synapses in the IHC and stereociliary bundle in the OHC exhibited the same longitudinal trend, i.e., the damage was observed in the basal cochlear region in response to LISW (high-frequency dominant shock wave), and in the entire cochlear region in response to the shock tube (low-frequency dominant shock wave). The peak forces potentially affect the high-frequency dominant cochlear damage especially in the LISW group. However, by using a stronger LISW than that in the present study, broader damage toward the cochlear apex was introduced as reported previously^29^. This previous report also indicates that the blast delivery method would be less involved in this frequency distribution. Therefore, we strongly suggest that the frequency-distribution pattern of the exposed blast would be the biggest factor to determine the distribution of cochlear damage.

Conversely, although a clear relationship was observed between the physiological (obtained with the ABR) and histological findings, the trend in the DPOAE after blast exposure was not clearly commensurate with the histological damage in the OHC at lower frequencies. The DPOAE can directly measure OHC function, which acts as a biological motor for amplifying the motion of the sensory epithelium^24^. However, loss of OHC was not observed in both groups in this research, whereas only a disruption of the OHC stereociliary bundle was observed, which could be the reason why the differences in DPOAE elevation could not be detected.

### The synapse in the IHC and stereociliary bundle in the OHC are the cochlear structures vulnerable to blast exposure

We confirmed the degeneration of the synapses in the IHCs and disruption of the stereociliary bundle in the OHCs, whereas there was no reduction in the number of the hair cells in the spiral ganglion after the blast exposure used in this research. The reduction in the synapses without hair cell loss is compatible with the reduction in the ABR wave I amplitude after LISW exposure because synaptic ribbon counts in normal ears provide an accurate metric of the afferent innervation of the IHCs^30^. This type of hearing dysfunction is described as cochlear synaptopathy^31^, which is revealed to be closely related to the pathogenesis of tinnitus^32^ and hyperacusis^33^. Conventionally, animal models of acute cochlear synaptopathy are created by noise exposure^34^; however, the damaged area is generally limited to the cochlear basal turn. By using different shock waves in the current study, we could easily induce synaptopathy in the whole cochlear area. Cochlear synaptopathy has recently been discovered in various types of SNHL, including age-related hearing loss and congenital/genetic hearing loss^35,36^. Therefore, we believe that our blast-induced hearing loss models would be valuable both in blast research and for the investigation of various types of hearing loss, whose pathogenesis involves cochlear synaptopathy. However, it needs to be mentioned that our presenting models did not purely constitute cochlear synaptopathy, as the relationship between the ABR amplitude and number of synapses is not correlated when threshold shift or HC damage occurs.

The stereociliary disturbance without hair cell loss observed in this study is a unique characteristic of blast-induced hearing dysfunction, which was also observed in our previous study using LISW^19^. We used confocal microscopic analysis using immunohistochemistry for the stereociliary bundle in addition to the conventional SEM in this study, as it is difficult to eliminate the artifacts in the bundle structures arising from their deformation by the vapor deposition process^37,38^. Therefore, we obtained the same result with both confocal scanning and SEM, and we believe that we were able to count the exact ratio of the disrupted bundles. We strongly suggest that the stereociliary bundle disruption was the main pathology, which caused an ABR threshold shift without any hair cell loss. However, various ratios of the disrupted stereociliary bundle were observed in the cochlear apical part even in the control group. The OHC hair bundle at the apical part is structurally wider and larger than that at the basal turn^39^; therefore, the stereociliary bundle at the apical turn is more fragile. This is the most plausible reason for the lack of a significant difference between the ratios of the disrupted bundles of the shock-tube and LISW groups, especially in the lower-frequency regions.

In this study, we did not observe the loss of the IHCs, OHCs, and SGCs in any group. The hair and ganglion cells may be more resilient to shock waves compared to the synapses or stereociliary bundles. Indeed, the loss of both types of hair cells^15,29^ and SGCs^13,19^ could be induced by using more intense shock waves generated by the shock tube or LISW. We could easily control the intensity of the shock wave (shock tube or LISW) using our shock-wave generator system. This adjustability of the shock-wave generator is an advantage of our system.

### Application of blast-induced hearing loss animal models: optimal species or shock-wave generator

The chinchilla model of hearing loss has been widely used for a long time, partially due to its human-like hearing range, low-impedance tympanic membrane, and large ear canal^15,40^. However, the mouse is the most widely used experimental animal due to its size, cost-effectiveness, and availability of transgenic variants. The physiological findings from LISW seem to be similar to hearing impairment involving a high-frequency hearing loss in humans caused by blast exposure that is encountered in clinical situations (based on the comparisons of the two shock-wave groups)^9,10^. The shock wave generated by our shock-tube chamber is low frequency-dominant for mice owing to the size of the cochlea (whereas it lies in the middle audible range for humans or chinchillas). From this perspective, LISW would be a suitable stimulation to generate mouse models of high-frequency blast-induced hearing loss, akin to the pathological state in humans.

In this study, we created a blast-exposed mouse model without TMP because we aimed to analyze the pure sensorineural component of hearing loss. However, TMP is one of the most frequently observed symptoms in patients exposed to real explosions^9,41^. Recent research has revealed that tympanic membrane rupture could limit the damage to the inner ear by absorbing a part of the shock wave’s energy^15^. Our LISW model could not induce TMP, because the shock wave (even the high-intensity ones) was irradiated directly to the inner ear^19,29^. A high-intensity blast generated using a shock tube would be the ideal shock-wave generator to analyze mixed hearing loss with TMP and SNHL. It is possible to choose the type of blast depending on the use or aim of the research. One of the limitations of this research is that the total energy of exposed blasts induced by different generators was not standardized. In this research, we prioritized the degree of damage at 16 kHz, a low-hearing-threshold frequency in mice, to facilitate the comparison of the effect of the two different blasts on the inner ear. To compare these two different blast delivery settings, standardizing the total energy of blast pressure would be needed.

The development of therapeutic strategies for blast-induced hearing loss is an urgent issue. The treatment modalities for blast-induced hearing loss involving cochlear synaptopathy or stereociliary bundle disruption can be applied to other types of SNHL with the same underlying pathology. As mentioned above, our blast-induced hearing loss models also represented cochlear synaptopathy, which is a good target for SNHL treatment^42^. Recent studies have revealed that neurotrophin-3^43^ and Rho-associated coiled-coil containing protein kinase inhibitor^44^ confer protective or regenerative effects against the loss of ribbon synapses after inner ear damage. Moreover, the disruption of the stereociliary bundle is also an important area of focus for SNHL, including noise-induced hearing loss. Forced expression of the Atoh1 gene reportedly has a regenerative function after the loss of stereociliary bundles produced by noise-induced hearing loss^45^. Our multiple blast-induced hearing loss models would be ideal platforms for research on various types of SNHL.

## Methods

### Animals

Thirty CBA/J mice (male, 6 weeks old) weighing 17–20 g were purchased from Charles River Laboratories (Yokohama, Japan). They were given free access to water, a regular diet, and were individually housed and maintained at 23–25 °C. All experimental procedures were approved by the Institutional Animal Care and Use Committee of the National Defense Medical College and conducted per the guidelines of the National Institutes of Health and the Ministry of Education, Culture, Sports, Science and Technology of Japan (approved #18,050). All experiments were performed per the ARRIVE guidelines (http://www.nc3rs.org.uk/page.asp?id=1357). All efforts were made to minimize the number and suffering of the animals.

### Blast exposures

#### Shock tube

Our shock tube constituted stainless-steel tubing (inner diameter: 25 mm, outer diameter: 34 mm), divided by a polyester diaphragm into high-and-low pressure parts (length of the low-pressure and high-pressure parts: 800 mm and 400 mm, respectively) (Fig. 1. a, b). Compressed nitrogen gas was driven into the high-pressure portion; the shock wave was propagated to the low-pressure portion by rupturing the diaphragm. Each mouse was placed outside the shock tube at 55 mm between the end of the low-pressure portion and the mouse’s ear canal. The shock wave was irradiated to the front of each mouse’s head from diagonally upward to prevent a blast wind (Fig. 1). The shock wave’s pressure profiles were measured using a microphone preamplifier (#426B03 PCB piezotronics). The output was recorded and converted using an oscilloscope (Power lab system 2/26 #ML826, AD instruments Inc, Depew, NY) with a 100-k/s sample rate. Data analysis was performed with the Scope 3 software (version 3.9.2, AD instruments Inc, Castle Hill, Australia). The peak pressure of the shock wave was set to 25 kPa, as it did not cause brain hemorrhage in the same shock tube model^18^.

#### Laser-induced shock wave (LISW)

LISWs were generated as described previously^19^: a laser target was irradiated with a 532-nm Q-switched neodymium-doped yttrium aluminum garnet (Nd: YAG) laser (Brilliant b, Quintal, Les Ulis Cerdex, France; pulse width, 6 ns) (Fig. 1. c, d). Specifically, the laser target was a 10-mm in diameter, 0.5-mm thick black natural rubber disk, and a 1.0-mm thick transparent polyethylene terephthalate sheet was bonded to the top of the target area to confine the laser-induced plasma, by which the LISW impulse was increased. The laser pulse was focused onto a 4.0-mm diameter spot on the laser target using a plano-convex lens. Output signals were recorded using a digital oscilloscope (DPO4104B, Tektronix, Tokyo, Japan; bandwidth, 1 GHz) and the signals were calibrated using the software provided by the manufacturer of the pressure sensor. The laser fluences were set at 2.0 J/cm^2^ to generate a similar degree of ABR threshold shift at that observed in the shock tube model at 16 kHz, as described in a previous study^19^.

#### Cochlear function tests

Immediately after the blast exposure, we observed the tympanic membrane using a small digital endoscope (AE-C1 endoscopic system, AVS Co., LTD, Tokyo, Japan) to examine the TMP on both ears.

Cochlear function tests were conducted for each animal at 6 log-spaced frequencies (half-octave steps from 5.6 to 32.0 kHz) before and 1, 7, and 28 days after blast exposure. The mice were anesthetized with ketamine (75 mg/kg, intraperitoneally) and medetomidine hydrochloride (1 mg/kg intraperitoneally). Needle electrodes were inserted at the vertex and pinna to detect the ABRs, with a ground electrode placed near the tail. The ABRs were evoked using 5 ms tone pips (0.5-ms rise-fall with a cos2 onset envelope delivered at 35/s). The response was amplified, filtered, and averaged using a LabVIEW-driven data-acquisition system (https://www.masseyeandear.org/research/otolaryngology/eaton-peabody-laboratories/engineering-core). The sound level was raised in increments of 5 dB from ≥ 10 dB below the threshold to < 80 dB sound pressure level (SPL). At each sound level, 1024 responses were averaged with alternated stimulus polarity. The “ABR threshold” was defined as the lowest SPL level at which any wave could be detected on visual inspection of the tacked waveforms, usually corresponding to the level just below that at which the peak-to-peak response amplitude rose significantly above the noise floor. When no response was observed at the highest available sound level, the threshold was designated as being 5 dB greater, to allow statistical tests. For amplitude versus level functions, the wave I peak was identified by visual inspection at each sound level and the peak-to-peak amplitude was computed.

To measure DPOAEs at 2f1–f2, the primary tones were set such that the frequency ratio (f2/f1) was 1.2 and the f2 level was 10 dB below the f1 level. For each f2/f1 primary pair, the levels were swept in increments of 5 dB from 20 to 80 dB SPL (for f2). Waveform and spectral averaging were used at each level to increase the signal-to-noise ratio of the recorded ear-canal sound pressure, and the amplitude of the DPOAE at 2f1–f2 was extracted from the averaged spectra along with the noise floor at the spectrum’s adjacent points. The threshold was defined as the f1 level required to produce a DPOAE at 0 dB SPL.

#### Cochlear processing

All histological examinations were conducted after the final ABR assessment (28 days after blast exposure). For whole-mount preparation, the mice were transcardially perfused with heparinized phosphate buffer saline (PBS) followed by 50 mL of 4% paraformaldehyde (PFA) in 0.1 M phosphate buffer (PB) at room temperature (20 °C–25 °C) under general anesthesia. After decapitation, the cochleae were quickly dissected and small openings were created at the round and oval windows, and the cochlea apex, which was then bathed overnight in 4% PFA in 0.1 M PB at 4 °C. After decalcification with 0.5 M ethylenediaminetetraacetic acid (EDTA) in PBS [Decalcifying Solution B (EDTA method); Wako Pure Chemical Industries Ltd., Osaka, Japan] for 4 days at 4 °C with shaking, each cochlea was microdissected into six pieces for whole-mount preparation.

The immunostaining protocol was performed according to the previous studies^15^, initiated by blocking the tissues for 1 h with 0.1% Triton X-100 in PBS supplemented with 5% normal goat serum to detect the hair cells and synapses. The fixed and permeabilized pieces were incubated overnight at 37 °C with a rabbit polyclonal antibody to mouse (IgG1) anti-CtBP2 at a 1:200 titer (#612,044, BD Transduction Labs), mouse (IgG2a) anti-GluR2 antibody at 1:2000 (#MAB397, Millipore) and rabbit anti-Myosin 7a at 1:200 (#25-6790 Proteus Biosciences). The cochlear pieces were washed, and the following secondary antibodies were incubated for 2 h at 37 °C: Alexa Fluor 488-conjugated goat anti-mouse (IgG2a) at 1:1000 (#A21131, Life Technologies), Alexa Fluor 568-conjugated goat anti-mouse (IgG1) at 1:1000 (#A21124, Life Technologies), and Alexa Fluor 647-conjugated chicken anti-rabbit at 1:200 (#A21443, Life Technologies). All primary and secondary antibodies were diluted in 1% normal horse serum with 0.3% Triton X-100. The immunostaining protocol for stereocilia analysis was also based on a previous study^15^. The tissue was permeabilized by freezing on dry ice, incubated for 1 h at room temperature in a blocking solution with 5% normal donkey serum in PBS and 0.3% Triton X-100 for 2 h at room temperature. The primary antibody, rabbit anti-espin 1:100 (ESPN, Sigma #MAB397), was incubated overnight at 37℃, and the secondary antibody, i.e., biotinylated donkey anti-rabbit FAB fragment 1:400 (Jackson ImmunoResearch #711-067-003), was incubated for 2 h at 37 °C. Following tertiary labeling, streptavidin-conjugated Alexa Flour 568 1:200 (Jackson ImmunoResearch #S-11226) was incubated for 1 h at room temperature. All antibodies were diluted in 1% normal donkey serum with 0.3% Triton X-100.

The specimens were washed thrice with PBS and mounted on slides containing an antifade medium (VECTASHIELD; Vector Laboratories, Burlingame, CA, USA) and viewed under a fluorescence microscope (BZ-X700, Keyence Corporation, Osaka, Japan) or confocal fluorescence microscope (for the observation of the stereocilia, TCS SP8, Leica, Wetzlar, Germany). The cochlear lengths were measured for each animal, and a cochlear frequency map was computed to precisely localize the IHCs in the 5.6, 8, 11.3, 16, 22.6, and 32 kHz regions, respectively. PhotoShop CC (Adobe, San Jose, CA), ImageJ software (NIH, Bethesda, MD), and ImageJ Plugin (http://www.masseyeandear.org/research/otolaryngology/investigators/laboratories/eaton-peabody-laboratories/epl-histology-resources/imagej-plugin-for-cochlear-frequency-mapping-in-whole-mounts) were used to measure the total length of the cochlear whole mounts and the length of the individual segments.

The mice were transcardially perfused with heparinized PBS followed by 4% PFA in 0.1 M PB at room temperature under general anesthesia to obtain the frozen cross-sections. After decapitation, the cochleae were quickly removed and bathed in 4% PFA in 0.1 M PB at 4 °C overnight. The specimens were first decalcified with 0.5 M EDTA in PBS for 14 days at 4 °C with shaking, and subsequently cryoprotected in a sucrose gradient (10%–30%) and embedded in an optimal cutting temperature compound for cryosectioning. Serial frozen sections of 10 µm were cut along a horizontal plane parallel to the cochlear modiolus, stained with hematoxylin and eosin, and viewed under a microscope (BZ-X700).

#### Scanning electron microscopy

The specimens for scanning electron microscopy (SEM) were obtained thusly: the mice were transcardially perfused with 0.01 M PB (pH = 7.4) containing 8.6% sucrose under general anesthesia, followed by fixation with freshly depolymerized 2% paraformaldehyde and 2.5% glutaraldehyde in 0.1 M PB (pH = 7.4) containing 5% sucrose. After decapitation, the cochleae were quickly removed, creating small openings in the round and oval windows, and apex of the cochlea, which were bathed overnight in the same fixative at 4 °C. After decalcification with 0.5 M EDTA in PBS [Decalcifying Solution B (EDTA method); Wako Pure Chemical Industries Ltd.) for 4 days at 4 °C with shaking, each cochlea was microdissected into six pieces for whole-mount preparation. The tissues were subsequently fixed with 1% OsO_4_ at 4 °C for 30 min, dehydrated in ethanol, critical point dried with liquid CO_2_, sputter-coated with osmium, and examined under an electron microscope (JSM-6340F, JEOL Ltd., Tokyo, Japan) operated at 5.0 kV.

#### Histopathological analysis

OHCs and IHCs were counted using confocal microscopy conducted at 5.6, 8, 11.3, 16, 22.6, and 32 kHz, while focusing on the presynaptic ribbons in the basolateral portion of the IHCs; an oil-immersion 100 × or water-immersion 60 × objective and a 0.2-μm z-step were used. Z-stacks were acquired at three adjacent areas for each frequency region in each cochlea: each stack contained approximately 10 IHCs per row. The number of OHCs and IHCs per 200 µm was counted at each point described above. The densities of the OHCs and IHCs per 200 µm were calculated and compared at each site.

The numbers of synaptic ribbons in the IHCs (CtBP2-positive puncta) and glutamate-receptor patches (GluA2 puncta) per 200 µm were counted at 5.6, 8, 11.3, 16, 22.6, and 32 kHz. The number of synaptic ribbons and glutamate-receptor patches per IHC was calculated and compared at each site. The counts were performed by three different blinded individuals.

The number of spiral ganglion cells (SGCs) in the basal, middle, and apical-middle turns of the cochleae was counted. We calculated the average SGC density obtained from the following three sections: the section considered as the center of each frequency site and 20 µm before and after the center section.

Quantitative analysis of stereociliary bundle disruption in the OHCs was conducted by calculating the ratio of the disrupted stereocilia (number of disrupted OHC stereocilia/total number of OHC stereocilia) from each energy group (n = 5 for each group) using the confocal stereocilial immunohistochemistry images. The number of stereocilia per 100 μm was counted at the center of the 5.6, 8, 11.3, 16, 22.6, and 32-kHz areas. One or more rows of OHC bundles that were bent toward the lateral side, tangled, or lacking their base were designated as “disrupted.” The counts were performed by three different individuals who were blinded to the experimental groups to minimize bias.

### Statistical analysis

Statistical analyses were performed using Prism 5 (GraphPad software, Inc., La Jolla, CA). All data values for the thresholds and amplitude of hearing function tests, and histological analysis were compared using the two-way ANOVA, followed by Bonferroni correction for multiple comparisons. The data values for the threshold shift and amplitude ratio were compared using a 2-tailed, unpaired Student’s *t-*test. *p*-values of < 0.05 were considered statistically significant. The standard error (SE) of the mean was represented with error bars.

## Supplementary Information

Supplementary Information.

## Data Availability

The datasets generated during and/or analyzed during the current study are available from the corresponding author on reasonable request.
