# Supplementary material for: Effect of shock wave power spectrum on the inner ear pathophysiology in blast-induced hearing loss
Source: Sci Rep. 2021 Jul 19;11:14704. doi: 10.1038/s41598-021-94080-0 (PMC8289960; doi:10.1038/s41598-021-94080-0)
Supplement: Supplementary file 1 — Supplementary Information. [file 41598_2021_94080_MOESM1_ESM.pdf]

Supplemental figures

Supplemental Figure 1. (a,b) The tympanic membrane findings after blast exposure. Tympanic membrane perforations or hemorrhage were seen. (c, d) The tympanic membrane perforation (c), and middle ear hemorrhage (d) caused by a much stronger shock wave than that used in this research.

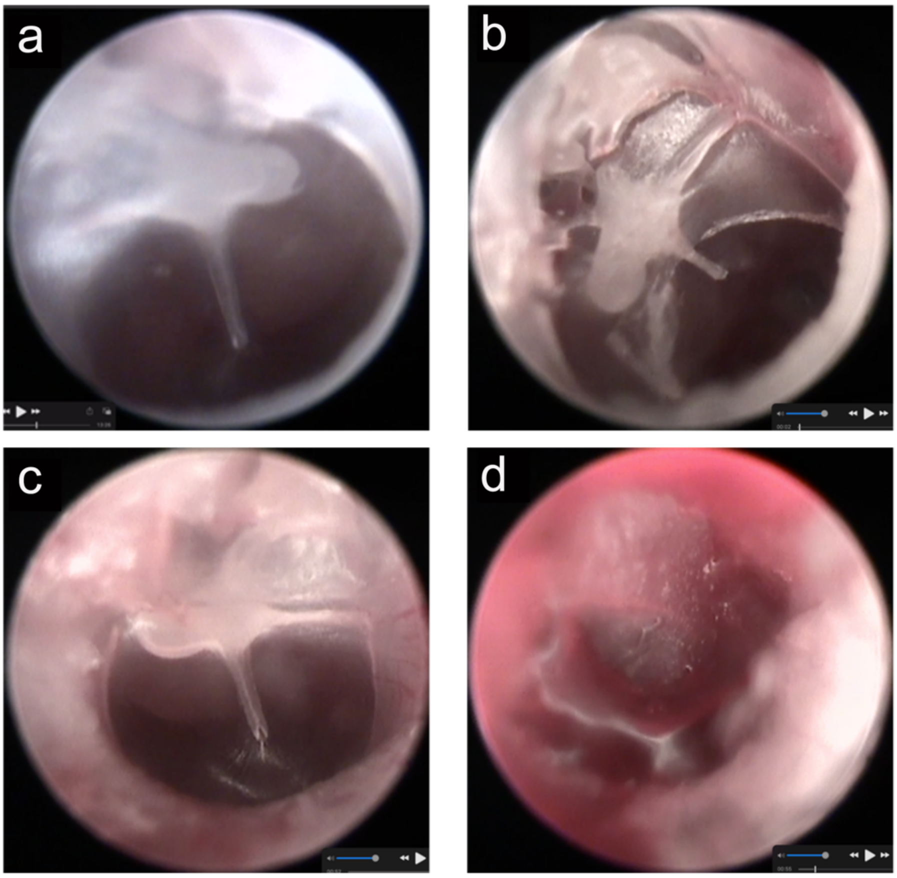

Supplemental Figure 2. Quantification analysis of the hair cells. There was no loss of IHCs (a) and OHCs (b) in the shock-tube and LISW groups.

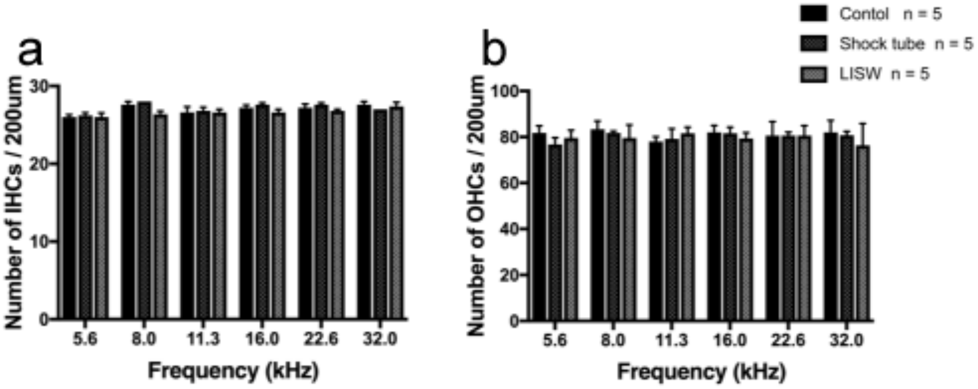

LISW: laser induced shock wave, OHC: outer hair cell, IHC: inner hair cell

Supplemental Figure 3. (a-c) Morphology of the spiral ganglion neurons with hematoxylin and eosin staining at middle turn of the cochleae. Neither loss nor degeneration of the spiral ganglion neurons were observed after shock-wave exposure in the shock-tube and LISW groups. (d) Quantification analysis. No significant decrease was observed in the number of spiral ganglion neurons in both groups.

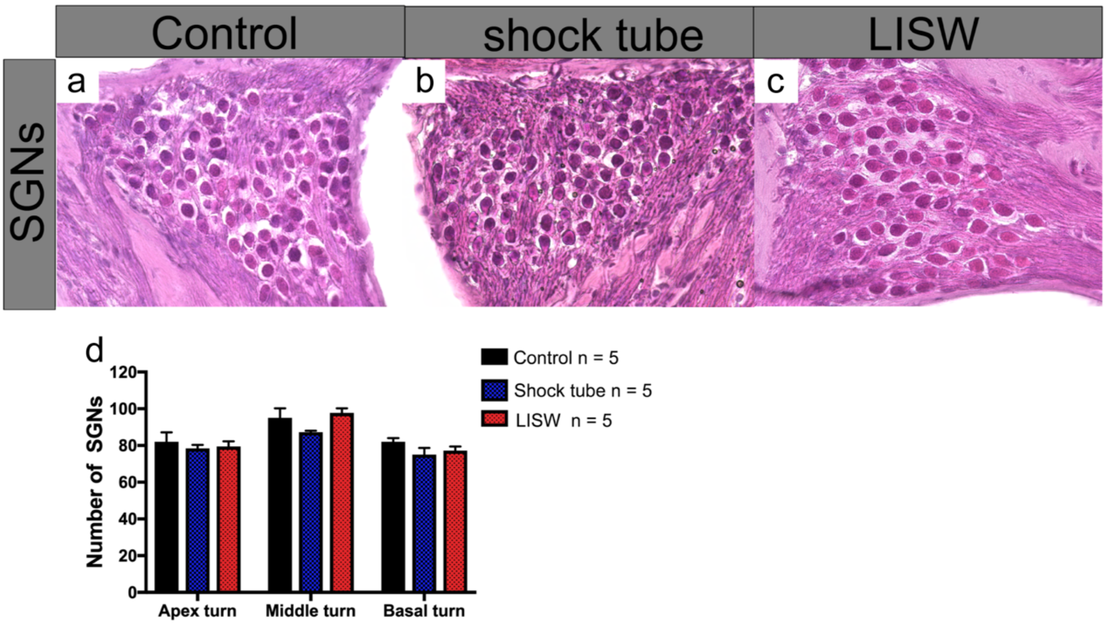

LISW: laser-induced shock wave
